# Effects of monoclonal antagonist antibodies on calcitonin gene-related peptide receptor function and trafficking

**DOI:** 10.1186/s10194-019-0992-1

**Published:** 2019-04-30

**Authors:** Raffi Manoukian, Hong Sun, Silke Miller, Di Shi, Brian Chan, Cen Xu

**Affiliations:** 1 Department of Cytometry Sciences, Amgen Research, 360 Binney Street, Cambridge, MA 02142 USA; 2 Department of Neuroscience, Amgen Research, 360 Binney Street, Cambridge, MA 02142 USA; 3 Department of Biologic Discovery, Amgen Research, 7990 Enterprise Street, Burnaby, BC V5A1V7 Canada; 4 Department of Neuroscience, Amgen Research, One Amgen Center Dr., MS 29-2-B, Thousand Oaks, CA 91320-1799 USA

**Keywords:** Calcitonin gene-related peptide, CGRP receptor, CGRP receptor antagonist antibody, Migraine, Receptor trafficking, Receptor recycling

## Abstract

**Background:**

Monoclonal antibodies against calcitonin gene-related peptide (CGRP) or its receptor are efficacious for the prevention of migraine headaches. The downstream molecular mechanisms following ligand-receptor blockade by which these antibodies prevent CGRP signaling through CGRP receptors have not been demonstrated.

**Methods:**

Here we produced tool monoclonal functional antagonist antibodies against CGRP and its canonical receptor and developed a novel cellular model using fluorogen-activated protein technology that allows detection of CGRP receptor internalization by flow cytometry and, for an extended time course, visualization by confocal microscopy.

**Results:**

Using this cell model we showed that these antagonist antibodies block both CGRP-induced cAMP signaling and CGRP receptor internalization. At least 10-fold higher concentrations of either antibody are necessary to block CGRP receptor internalization compared with cAMP accumulation in our cell model.

**Conclusion:**

These data reinforce our understanding of how monoclonal functional antagonist antibodies interfere with CGRP signaling.

## Background

Migraine is a highly prevalent neurological condition with immense socioeconomic impact on workplace productivity and quality of personal life [1]. Prophylactic therapy is recommended for many patients [2], but established drugs for migraine prevention have limited success due to inadequate efficacy, tolerability and patient adherence [3]. Novel monoclonal antibodies that target the neuropeptide calcitonin gene-related peptide (CGRP) or its receptor have been approved for use in migraine prevention and have consistently shown positive results in clinical trials (for recent reviews, see [4, 5]). The mechanism by which these monoclonal antibodies are proposed to prevent migraines is by blocking CGRP transmission in the trigeminovascular system, a key pathway involved in headache [6].

Monoclonal antibodies that target the neuropeptide CGRP (galcanezumab, eptinezumab and fremanezumab) are proposed to bind and thus deactivate CGRP released by trigeminal sensory nerve fibers [7], whereas antibodies that target the CGRP receptor (erenumab) presumably act by preventing access of CGRP to its canonical receptor [8]. There are two isoforms of CGRP: CGRPα is the predominant form in the central and peripheral nervous system implicated in migraine pathology and targeted by monoclonal antagonist antibodies. CGRPβ is mainly found in the enteric nervous system and is less well studied [9, 10]. The functional CGRP receptor comprises the G-protein coupled receptor calcitonin-receptor-like receptor (CLR) and receptor activity-modifying protein 1 (RAMP1) [11, 12]. Binding of CGRP to the extracellular binding pocket formed by the heterodimer causes activation of G proteins containing the Gαs subunit bound to CLR, which in turn activates adenylyl cyclase and cyclic adenosine monophosphate (cAMP)-dependent signaling pathways [9] that mediate vasorelaxation in blood vessels [13]. Following activation, the CGRP receptor and its ligand CGRP are internalized in a β-arrestin-dependent fashion and, depending on the temporal characteristics of the activation, are either recycled back to the cell membrane or degraded: Transient receptor activation leads to internalization followed by receptor recycling back to the cell surface and resensitization, while sustained activation leads to internalization and receptor degradation [14]. Internalization of the CGRP receptor into endosomes triggers a second wave of signaling, which is important for pain transmission [15].

To investigate the underlying mechanism of action of antagonistic monoclonal antibodies against the neuropeptide CGRPα (herein referred as CGRP) and the CGRP receptor, we produced human tool antibodies similar to the therapeutic antibodies used in clinical trials and tested these antibodies in a novel cellular model using fluorogen-activated protein (FAP) technology to assess functional cAMP production and CGRP receptor internalization dynamics. FAP-tagged proteins only fluoresce when bound by cognate activating fluorogens of different wavelengths, allowing for assessment of ligand-induced receptor dynamics and internalization [16, 17]. By combining FAP technology with flow cytometry and high resolution confocal microscopy, we were able to track subcellular localization of the CGRP receptor in the presence of CGRP and CGRP receptor antagonist antibodies in Chinese hamster ovary (CHO) cells in vitro. This novel cell model allowed us to explore and characterize the relationship between antagonistic monoclonal antibodies and CGRP-induced CGRP receptor internalization, a dynamic that until now has been unclear.

## Methods

### Study design

Recombinant Chinese hamster ovary K-1 cell lines (CHO-K1, ATCC, Manassas VA, USA, herein referred to as CHO cells) were generated expressing non-tagged and CLR-FAP- and RAMP1-his-tagged chimeras. A cAMP functional assay was used to confirm the activity of both the tagged and CLR-FAP tagged recombinant CGRP receptor complex in response to CGRP, a truncated CGRP receptor peptide antagonist CGRP_8–37_, which has been widely used in the literature [18, 19], and anti-CGRP (8E11) and anti-CGRP receptor (AA58) antibodies. The tool antagonist antibodies 8E11 and AA58 were generated at Amgen Inc. to recognize CGRP and CGRP receptor epitopes, respectively, similar to therapeutic antibodies used in clinical trials. FAP flow cytometry and high resolution confocal microscopy were then used to assess CGRP receptor subcellular localization after treatment with CGRP alone or in combination with the antagonist antibodies.

### Cell lines and cell culture

All cell culture reagents were purchased from Gibco (Thermo Fisher, Waltham, MA), unless otherwise specified. To assess localization of the CGRP receptor complex, we constructed CGRP receptor chimeras comprised of CLR fused to a Fluorogen Activating Protein (FAP)-tag and RAMP1 fused to a His-tag. These constructs were stably transfected into CHO cells to create tagged recombinant CGRP receptor expressing cells. CHO cells were transfected using a two-plasmid system with pSLX240.3puro HA-FAP-myc-CALCRL and pSLX240.2hygro H6GS3Ramp1 (Selexis, Sunnyvale, CA). Cells stably expressing the tagged CGRP receptor complex were grown in Ham’s F12 medium with 10% fetal bovine serum albumin (FBS) and 1X penicillin, streptomycin and glutamine (PSG), 400 μg/mL Hygromycin B (Sigma-Aldrich, St. Louis, MO) and 5 μg/mL Puromycin. CHO cells stably expressing untagged receptors were used as control for cAMP assay and FACS experiments. These cells were grown in Ham’s F12 medium with 10% FBS and 1X PSG, 0.4 mg/mL G418 (Geneticin) and 10 μg/mL Blasticidin (Invitrogen). Non-transfected CHO parental cells were maintained in Ham’s F12 medium with 10% FBS + 1X PSG and used as specificity control for the antibodies used for immunocytochemistry.

### Antibody generation

*AA58:* AA58 is a fully human antibody that recognizes the CGRP binding site composed of the extracellular domains of CLR and RAMP1 [20]. AA58 was generated by immunization of XenoMouse® animals (Amgen Inc., Burnaby, CA, USA) with purified soluble CGRP receptor protein as the antigen as described by Shi, et al. [20] In brief, 293-6E cells were transiently co-transfected with the N-terminal extracellular domains of human CLR (amino acids 1–138 of GenBank accession no. AAA62158) and human RAMP1 (amino acids 1–117 of GenBank accession no. CAA04472) to generate soluble CGRP receptor polypeptides. A pool of mice with the highest sera titer was used to generate hybridomas using a standard protocol [21] and AA58 was identified through competitive binding, functional antagonism and selectivity assays at the human CGRP receptor. The specificity of AA58 as a tool for detecting the CGRP receptor complex using immunofluorescence techniques has previously been demonstrated [20].

#### 8E11:

The fully human monoclonal antibody 8E11 against CGRPα was generated by immunizing transgenic mice (XenoMouse® animals) using a conventional immunization method (Amgen Inc., Burnaby, CA, USA). The mice received four rounds of immunizations every two weeks with soluble human CGRPα conjugated to peptides representing T-cell epitopes (TCE peptides). Mice were immunized subcutaneously at the base of tail and intraperitoneally with up to 10 μg of antigen emulsified with TiterMax® Gold (Norcross, GA, USA). Mice with the highest detected enzyme-linked immunosorbent assay (ELISA) titer to soluble human CGRPα were selected for harvest and subsequent fusion. Four days prior to lymph nodes and spleen harvest, mice were given a final base of tail and intraperitoneal boost with 5 μg of soluble human CGRPα conjugated to TCE peptides in phosphate buffered saline (Cat# SH30256.02, GE Healthcare HyClone, Chicago, IL, USA). Hybridomas were then generated using standard techniques [22], plated onto 96 well culture plates and cultured for 2 weeks to generate exhausted supernatant for screening. 8E11 was identified through screening assays including binding, competition, binning, inhibition, kinetics and selectivity against human CGRPβ.

#### Animal care

Mice were housed under specific pathogen-free conditions at the Amgen Laboratory Research Facility and certified by the Canadian Council on Animal Care in strict regulations with associated standards and policies. The protocol was approved by the Animal Care Committee of Amgen British Columbia. Animals were group-housed on corn cob bedding and have ad libitum access to food and water via an automatic watering system. Animals were maintained on a 12:12 h light:dark cycle in rooms at constant temperature and humidity.

### cAMP functional assay

Functional effects of CGRP, the monoclonal antibodies AA58 and 8E11, and the truncated peptide antagonist CGRP_8–37_ were assessed in both recombinant CHO cells stably expressing untagged or CLR-FAP-tagged CGRP receptors using a cAMP assay (LANCE® Ultra cAMP, Perkin Elmer). Recombinant CHO cells expressing tagged or untagged CGRP receptors were added to 96-well half-area white plates (2000 cells per well) and were treated with CGRP at concentrations from 0.5 pM to 100 nM and with AA58, 8E11 and CGRP_8–37_ at 5pM to 10 μM to assess agonist activity. To assess antagonist activity, AA58, 8E11 and CGRP_8–37_ (positive control) were added to cells at 0.5 pM to 1 μM for 30 min at room temperature. 20 pM of CGRP corresponding to the EC_70_, was then added and cells were further incubated for 15 min at room temperature. The reaction was stopped by adding detection mix (LANCE® Ultra cAMP, Perkin Elmer) to all wells followed by a 45-min incubation at room temperature. The assay plates were read on an EnVision® Multilabel Plate Reader (PerkinElmer; Waltham, MA, USA) at an emission wavelength of 665 nm. The maximal cAMP accumulation response (considered 100% of control) was measured from wells containing agonist only (the highest concentration of CGRP for agonist curves). Measurements from wells also containing antagonist were normalized to the maximal control value and expressed as percentage of control (POC). Wells containing assay buffer without test compounds were considered 0%. All results were analyzed by nonlinear regression curve fit using GraphPad Prism version 6 (GraphPad Inc., La Jolla, CA) and data are presented as mean ± SD. Data from the agonist dose-response curves were used to calculate half maximal effective concentration (EC_50_) and half maximal inhibitory concentration (IC_50_) values for agonist and antagonist studies, respectively.

### Flow cytometry

CHO cells expressing FAP-tagged CGRP receptor were dissociated and resuspended in the media described above at 2 million/mL. 10 nM of βRed fluorogen was added to the cell suspension and incubated for 2 min. 100 µL of cell suspension was added directly to wells of a 96 well plate containing 100 µL of 2X CGRP, AA58 or 8E11. Cells were incubated for 35 min in 37°C/5% CO_2_ and then cooled on ice for 5 min to stop reaction. 50 µL of βGreen fluorogen was added to the cells for a final concentration of 150 nM. Plates were incubated at room temperature for 2 min. Plates were directly assayed by flow cytometry using a BD Bioscience HTS sampler unit connected to a BD Bioscience LSR II flow cytometer (BD Bioscience, San Jose, CA, USA). 488 nm laser line was used to excite the βGreen fluorogen (membrane-bound CLR) and a 561 nm laser to excite the βRed (internalized CLR). Healthy cells were determined and gated using the size (forward scatter) and complexity (side scatter) parameters. Gated cells were measured for their mean fluorescence intensity, which were collected for each sample and analyzed using DIVA™ software (BD Bioscience).

### CGRP induced internalization using immunofluorescence detection

Immunofluorescence and confocal microscopy were used to identify and characterize the membrane bound versus subcellular localization of the CGPR receptor.

Cells were cultured to sub-confluency in a 96-well, black-walled plate with optical grade glass bottoms (PerkinElmer, Waltham, MA). For sustained exposure, media containing 100 nM CGRP was added to washed wells, over a time course of up to 24 h. Cells were processed (described below) at 0, 5, 10, 60 min and 5 h and 24 h. For transient exposure, the CGRP containing media was removed after 5 min and cells remained in culture over the 24-h time course, sampled at the same time points above. AA58 or 8E11 (each at 70 μg/mL; 479 nM) were either added alone at the time points above or added at the same time together with 100 nM CGRP and incubated in sustained or transient mode as described above. For immunofluorescence staining, cell culture media was removed from plate wells, cells were washed with wash buffer (phosphate buffered saline (PBS)/0.5% bovine serum albumin (BSA)), and subsequently fixed by overlaying 10% neutral-buffered formalin (Sigma) for 15 min at room temperature. After two washes of 5 min each with wash buffer (PBS/0.5% BSA), cells were permeabilized with 0.1% Triton X-100 in PBS for 20 min, washed twice again for 5 min and then 100 μL of desired antibody cocktail was added: AA58 (5 μg/mL), 8E11 (5μg/ml) and RAMP1 rabbit monoclonal (EPR10867, Abcam, Cambridge, MA, USA; 1:1000), were used to detect the CGRP receptor [20], ie, the CGRP binding site at the interface of CLR and RAMP1, respectively. Staining of untransfected parental cells, which lack the CGRP receptor, with AA58 or RAMP1 antibody was used as negative control. A mouse monoclonal antibody against early endosome antigen 1 (EEA1, 610,457, BD Biosciences, Franklin Lakes, NJ, USA, 5 μg/mL) was used to label early endosomes. LAMP2 is one of the lysosome-associated membrane glycoproteins [23, 24]; therefore, LAMP2 mouse monoclonal antibody (ab25361, Abcam, Cambridge, MA, USA, 5 μg/mL) was used to identify lysosomes. Secondary antibodies Rabbit IgG1 polyclonal AlexaFluor546 (ThermoFisher, Waltham, MA, USA; 1:1000) and Goat anti-human IgG Fc-FITC (Novex, Life Technologies, Waltham, MA, USA; 1:1000) were used with appropriate primary antibodies; primary antibodies were incubated overnight at 4°C and secondary antibodies for 2 h at room temperature; FAP fluorogen βRed membrane impermeant was used at 1:1000 prior to fixation (Spectragenetics, Pittsburgh, PA). The final wash buffer contained 2 μg/mL Hoechst (ThermoFisher, Waltham, MA) solution in PBS as a nuclear counterstain. Concurrent AA58 and 8E11 immunostaining could not be used due to potential cross reactivity of two human primary antibodies with an anti-human secondary antibody.

### Confocal microscopy

High-content imaging was performed on a PerkinElmer Ultraview Vox spinning disc confocal microscope (PerkinElmer, Waltham, MA, USA) using laser excitation wavelengths of 405 nm, 488 nm, 561 nm and 633 nm. A Nikon Plan Fluor 40x/0.75 and Apo TIRF 60x/1.49 oil were used for image capture. Single plane optical slice images were taken and represent a thickness of approximately 0.15 µM. Images were acquired using the PerkinElmer Volocity software 6.3 (Perkin Elmer), where some contrast enhancement was used to enhance viewing. Images were exported to Photoshop Elements 2.0 (Adobe Inc. San Jose, CA) where they were formatted for publication.

## Results

### Expression of functional CGRP receptors in recombinant CHO cells

**Fig. 1:**
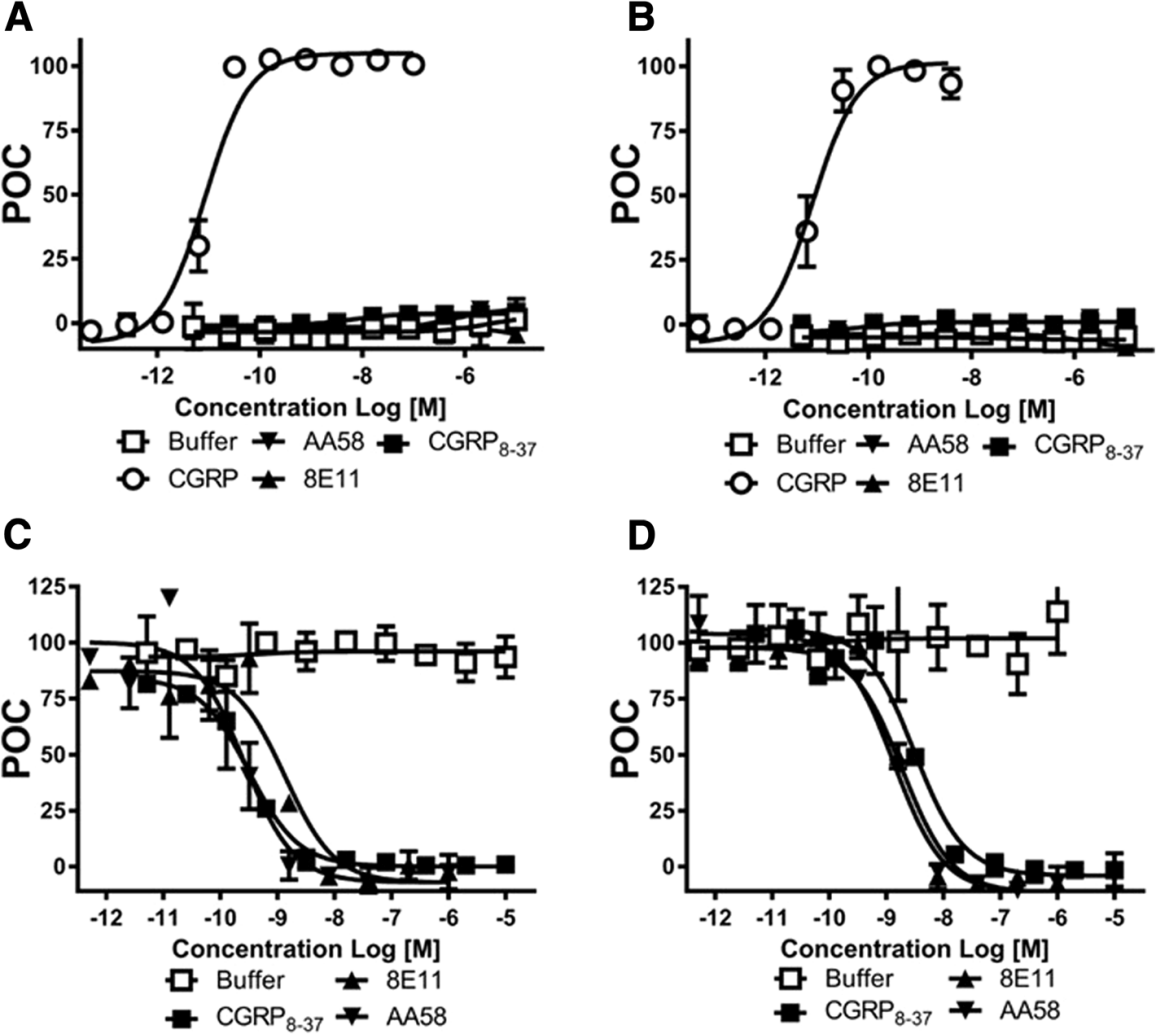
CGRP-stimulated cAMP production. CHO cells stably expressing un-tagged (**a**, **c**) and CLR-FAP-tagged (**b**, **d**) CGRP receptors were treated with increasing concentrations of CGRP, AA58, 8E11 or CGRP_8–37_ (**a**, **b**). cAMP luminescence was measured and displayed as a percent of control (POC). CGRP produced a dose-dependent increase in both un-tagged (EC_50_ = 8.5 pM, **a**) and CLR-FAP tagged (EC_50_ = 8.2 pM, **b**) CHO cells, whereas AA58, 8E11 and CGRP_8–37_ demonstrated no agonist activity in either cell line (**a**, **b**). Preincubation with AA58, 8E11 or CGRP_8–37_ inhibited CGRP-induced (20 pM) cAMP production to a similar degree in both un-tagged (IC_50_ = 0.3, 1.4 and 0.3 nM, respectively, **c**) and CLR-FAP tagged CHO cells (IC_50_ = 1.4 nM, 1.9 nM and 3.2 nM, respectively, **d**)

To examine the functionality of recombinant CGPR receptors, we compared intracellular cAMP concentrations in response to CGRP, CGRP_8–37_, anti-CGRP antibody 8E11 and anti-CGRP receptor antibody AA58 in both untagged (Fig. 1a) and CLR-FAP-tagged (Fig. 1b) CGRP receptor expressing cells. CGRP was tested at concentrations from 0.5 pM to 100 nM and AA58, 8E11 and CGRP_8–37_ at 5 pM to 10 μM. The EC_50_ of CGRP in FAP-CLR-tagged CGRP receptor cells was comparable to that in untagged CGRP receptor cells (8.5 pM and 8.2 pM, respectively) indicating that the FAP- and His-tags did not affect the binding and functionality of CGRP receptor. It is worth noting that CGRP is more potent in the current CHO cell model system when compared to the literature EC_50_ of ~ 100 pM in the Swiss3T3 or HEK293 cells [12], and in human neuroblastoma cells (SKN-MC) endogenously expressing human CGRP receptors (EC_50_ = 670 pM) [20]. The inconsistency is most likely attributed to different host cells, and a very high expression level of the receptor, designed purposefully to visualize the receptor trafficking in this study. AA58, 8E11, and the peptide antagonist CGRP_8–37_ demonstrated no agonist activity in either cell line (Fig. 1 a and b). Next, we measured the intracellular concentrations of cAMP in the presence of CGRP (20 pM, corresponding to the EC_70_) with increasing concentrations (0.5 pM to 1 μM) of the antagonists AA58, 8E11, CGRP_8–37_ in both, untagged (Fig. 1c) and CLR-FAP-tagged cells (Fig. 1d). CGRP-mediated cAMP production in FAP-CLR-tagged and untagged CGRP receptor expressing cells was inhibited by all agents in a dose-dependent fashion and the IC_50_s were comparable between the two cell lines: The IC_50_ of AA58 was 0.3 nM and 1.4 nM, of E811 1.4 nM and 1.9 nM, of CGRP_8–37_ 0.3 nM and 3.2 nM in untagged versus tagged CGRP receptor expressing cells, respectively (Fig. 1c and d).

### Inhibition of CGRP-induced CGRP receptor internalization

**Fig. 2:**
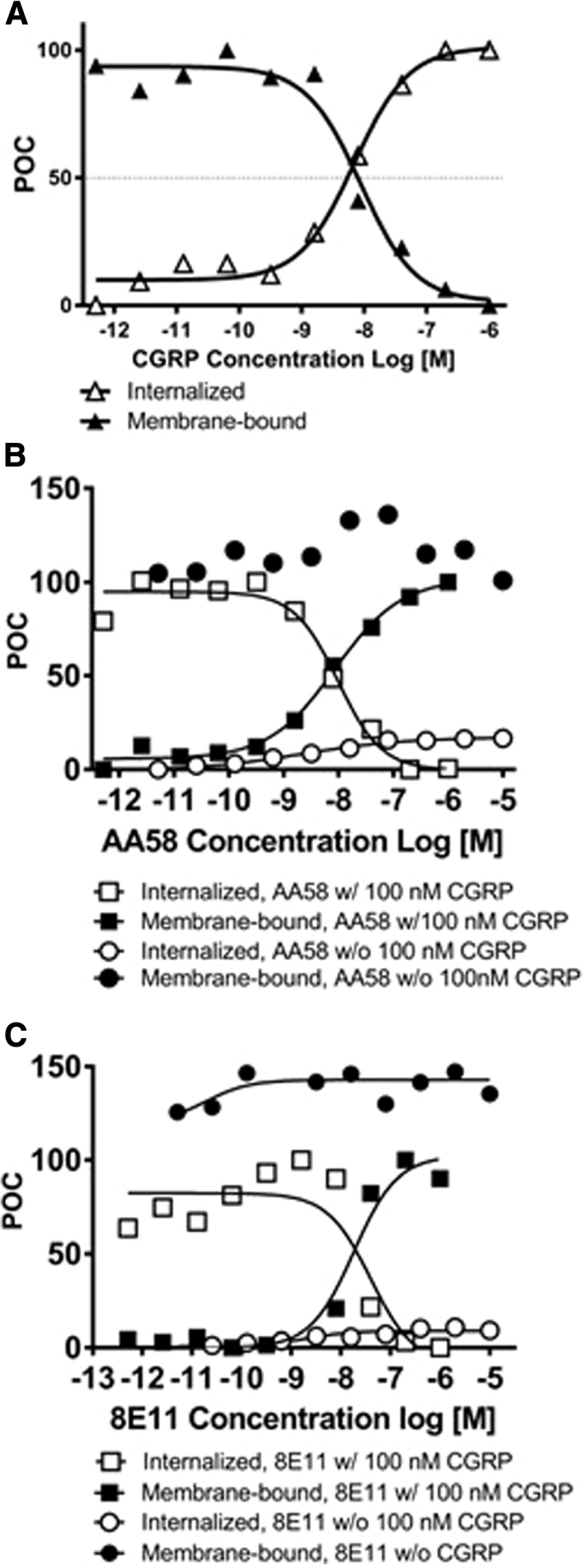
Inhibition of cAMP-induced CGRP receptor internalization. CLR-FAP tagged CGRP receptor internalization was measured using flow cytometry and graphed as percent of control mean fluorescence intensity (POC). Increasing CGRP concentrations resulted in CLR-FAP-tagged CGRP receptor internalization (open triangles) with a correlative decrease of membrane-bound CLR-FAP-tagged CGRP receptors (closed triangles) (**a**). In the presence of 100 nM CGRP, AA58 (**b**) or 8E11 (**c**) inhibited internalization (open squares) and concomitantly increased membrane-bound CLR-FAP-tagged CGRP receptors (closed squares), whereas no intrinsic effect was observed in the absence of CGRP (open and closed circles)

CGRP-induced receptor internalization dynamics were studied using the quantitative flow cytometry-based dual-signal FAP assay (Fig. 2). CGRP induced a dose-dependent internalization of the CGRP receptor as measured by FAP-tagged CLR localization, with a clear inverse relationship between internalized receptor (red signal, EC_50_ 7.0 nM) and non-internalized receptor (green signal, EC_50_ 8.8 nM) (Fig. 2a). CGRP-induced receptor internalization at 100 nM CGRP (corresponding to the EC_90_) was inhibited by AA58 with an IC_50_ of 9.8 nM and 8.7 nM for the internalized, red signal and uninternalized, green signal, respectively (Fig. 2b). Similarly, 8E11 inhibited CGRP-induced receptor internalization with an IC_50_ of 40 nM (internalized, red signal) and 18 nM (uninternalized, green signal) (Fig. 2c). There was no receptor internalization with AA58 or 8E11 in the absence of CGRP ligand indicating that antibody binding of the receptor or the neuropeptide itself does not induce CGRP receptor internalization and trafficking (Fig. 2b and c).

### Subcellular localization of the CGRP receptors

**Fig. 3:**
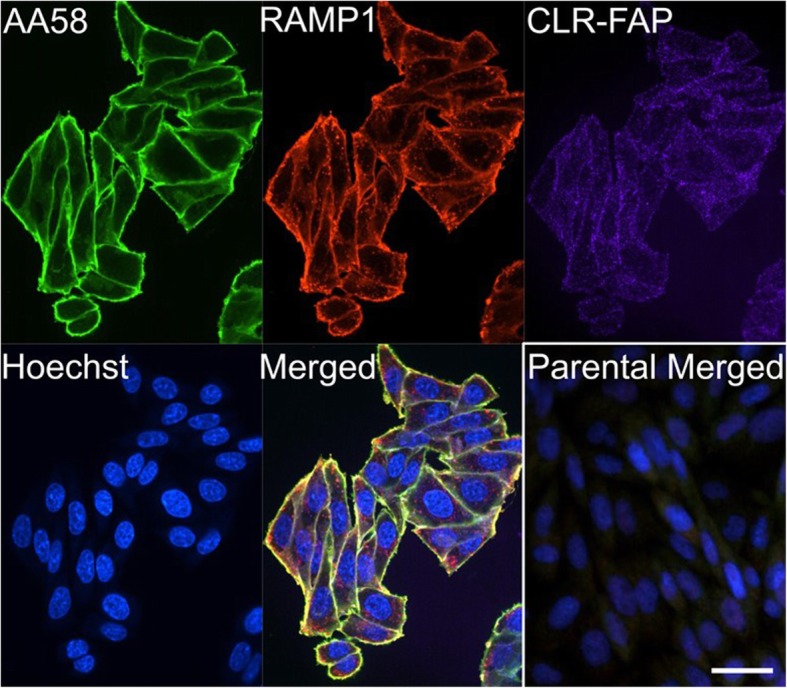
Colocalization of CGRP receptor subunits. AA58 (green) and RAMP1 (red) immunoreactivity co-localized (merged) with CLR-FAP fluorescence (magenta) as shown in a confocal optical slice of 0.15 μm with magnification 60x. Note the prominent membrane labeling by all three CGRP receptor detection agents. Additional, non-co-localized RAMP1 immunoreactivity (red) can be seen in the cytoplasm (merged), likely representing an artifact from overexpression. No immunoreactivity was observed in parental cells not expressing CGRP receptors (parental merged). Scale bar = 12 μm

We next used immunostaining and confocal microscopy to visualize and confirm co-localization of RAMP1 and CLR-FAP fluorescence in the CHO cell model (Fig. 3). AA58 immunostaining was used to visualize the CLR/RAMP1 heterodimer [20] and resulted in prominent membrane staining of FAP-CLR-tagged cells. Similarly, immunostaining with the RAMP1 antibody resulted mainly in labeling of the plasma membrane. Also, CLR-FAP fluorescence imaging resulted in a faint signal at the membrane. Immunoreactivity of both, AA58 and RAMP1 colocalized with CLR-FAP fluorescence suggesting that CGRP receptors are predominantly localized at the cell membrane of CHO cells. However, some RAMP1 immunoreactivity was seen in the cytoplasm and did not co-localize with AA58 immunoreactivity. This cytoplasmic, non-CGRP receptor-associated RAMP1 immunoreactivity may be an artifact resulting from overexpression, ie, there may be more RAMP1 expressed by the cells than can co-localize with the amount of CLR expressed. There was no FAP fluorescence nor immunoreactivity of either AA58 or RAMP1 detected in the parental CHO cells, which lack CGRP receptors. Together, these results suggest that AA58 and RAMP1 immunofluorescence staining and FAP imaging comprise a specific method for detecting the subcellular localization of the CGRP receptors in the recombinant CHO cell model.

### Time course of internalization of the CGRP receptors

**Fig. 4:**
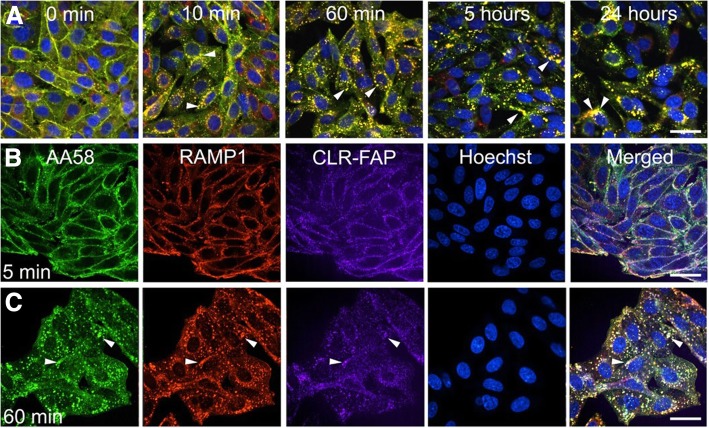
CGRP-induced receptor internalization. 24 h time course of CGRP (100 nM) induced internalization of CGRP receptors triple labeled by AA58, RAMP1 and CLR-FAP: Merged images show co-localization in yellow, nuclei are blue. Arrow heads point to endosomal-like vesicles that appear after 10 min of CGRP stimulation and continue to increase over the 24 h time points (**a**). Individual channels (AA58 = green, RAMP1 = red, CLR-FAP = magenta) are shown in a 0.15 μm optical slice at 5 min (**b**) and 60 min (**c**) after CGRP stimulation (CLR-FAP fluorescence is not visible at 0 min). Arrow heads point to endosomal-like vesicles. Scale bars = 12 μm

We next used the AA58 and RAMP1 staining, as well as CLR-FAP imaging described above to investigate trafficking of both CGRP receptor components individually and together in the presence of CGRP at 0–5 min, 10 min, 60 min, 5 h and 24 h fixed time point intervals (Fig. 4a). Hoechst staining (blue) of the nuclei confirmed cell health during the 24 h experiment. Even at the last time point observed, the cells did not present any obvious signs of apoptosis, based on normal nuclear morphology and lack of condensed DNA. At baseline (0–5 min), AA58, RAMP1 and CLR-FAP staining is mostly co-localized at the plasma membrane with few triple-positive, putative endosomal vesicles present in the cytoplasm of the cells (Fig. 4a and b). 100 nM CGRP, corresponding to the EC_90_ in FAP-tagged CHO cells based on the FACS experiment described before, induced a time-dependent increase of these endosomal vesicle-like structures that resulted in dense, triple-positive, perinuclear staining over time (Fig. 4a and c): As early as 10 min after CGRP application, there was pronounced formation of these triple-positive endosome-like vesicles (Fig. 4a). By 60 min, the plasma membrane was largely devoid of staining and most of the positive signal was observed in endosome-like vesicles (Fig. 4a and c). Between 60 min and 5 h, the triple-positive vesicular staining increased to span larger perinuclear areas (Fig. 4a). This staining pattern persisted in the cytoplasm up to 24 h, the last time point assessed. The strong triple-positive plasma membrane immunofluorescence seen at baseline did not reappear and was not observed up to 24 h, indicating absence of CGRP receptor recycling within the timeframe observed (Fig. 4a).

### Co-localization of internalized CGRP receptors with EEA1 and LAMP2

**Fig. 5:**
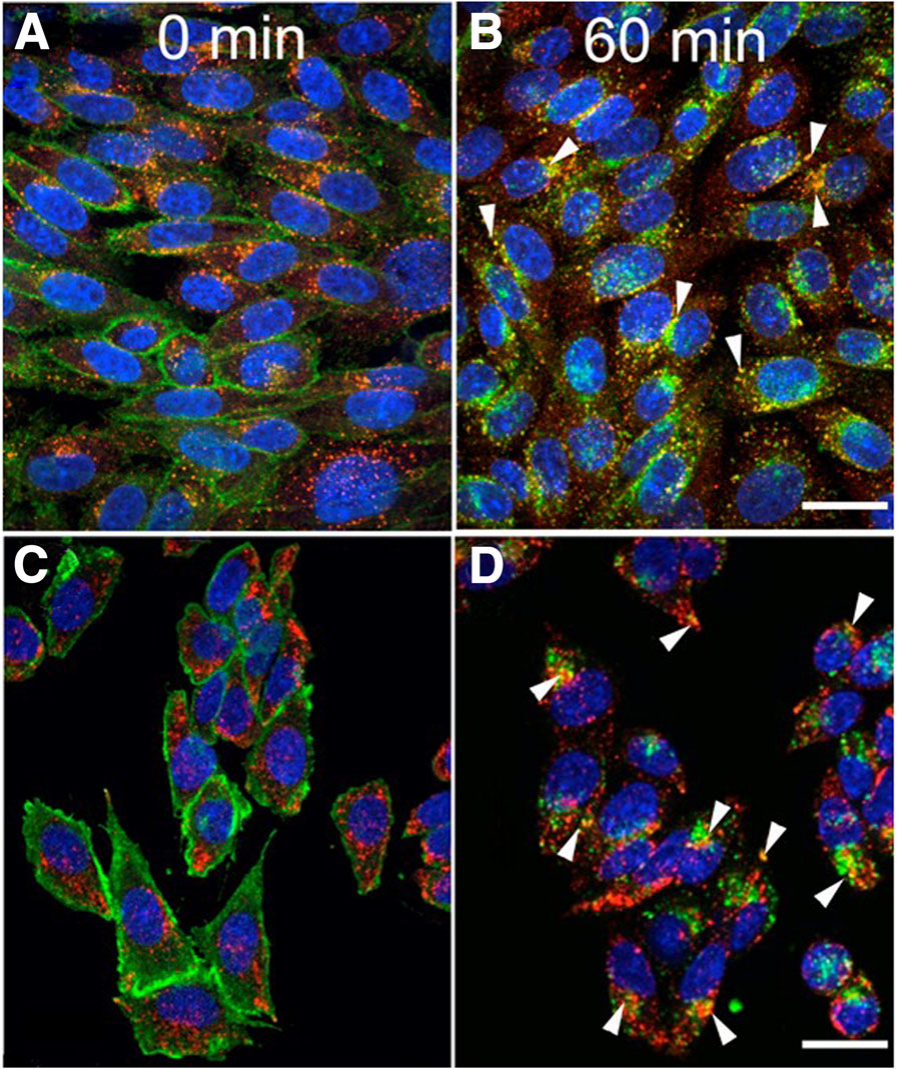
Colocalization of CGRP receptors with endosomes and lysosomes. CGRP receptors were stained with AA58 (green) and endosomes (**a**, **b**) with EEA1 (red) or lysosomes (**c**, **d**) with LAMP2 (red). Nuclei were labeled by Hoechst (blue). At 0 min, green AA58 immunoreactivity is localized to the plasma membrane, whereas red EEA1 (**a**) or LAMP2 (**c**) immunoreactivity is seen in vesicles throughout the cytoplasm (**a**). At 60 min after stimulation with 100 nM CGRP, the CGRP receptors are internalized and AA58 immunoreactivity is localized in vesicles in the cytoplasm (**b**, **d**). AA58 immunoreactivity is largely colocalized with EEA1 immunoreactivity (**b**, yellow, arrowheads). Only few of the vesicles at this time point are double positive for AA58 and LAMP2 (**d**, yellow, arrowheads), indicating that CGRP receptors are being degraded in lysosomes. Scale bars = 12 μm

To investigate whether the CGRP receptors that internalized after continuous CGRP exposure were associated with endosomes, we used an EEA1 antibody as an endosomal marker together with AA58 staining for the CGRP receptor and compared staining at 0 and 60 min after exposure to 100 nM CGRP (Fig. 5a and b). At 0 min, CGRP receptors labeled by AA58 were localized to the plasma membrane, whereas EEA1-positive endosomes were dispersed throughout the cytoplasm (Fig. 5a). At 60 min after CGRP exposure, CGRP receptors were internalized and AA58 immunoreactivity was largely co-localized with EEA1 immunoreactivity (Fig. 5b). To investigate whether some of the CGRP receptors were undergoing degradation through the lysosomal pathway as described previously [14], we used a LAMP2 antibody as a lysosomal protein marker together with AA58 staining for the CGRP receptor and compared staining at 0 and 60 min after exposure to 100 nM CGRP (Fig. 5c and d). At 0 min AA58 staining was confined to the plasma membrane, whereas LAMP2 labeled lysosomes in the cytoplasm (Fig. 5c). At 60 min after CGRP exposure, AA58 and LAMP2 co-staining started to appear, suggesting that some CGRP receptors were beginning to be degraded by lysosomes (Fig. 5d).

### Recycling of CGRP receptors after transient exposure to CGRP

**Fig. 6:**
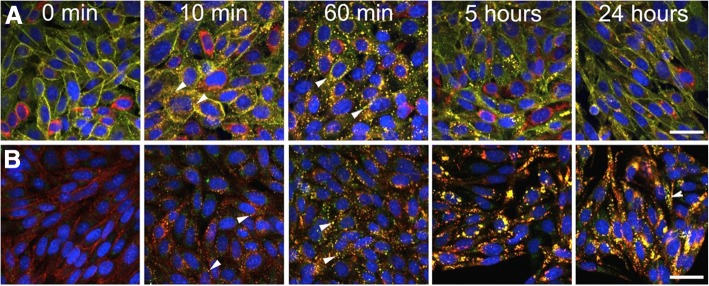
CGRP receptor and CGRP recycling. Time course of transient, 100 nM CGRP-induced internalization of CGRP receptors triple positive (yellow) for AA58 (green), RAMP1 (red), CLR-FAP (magenta) (**a**). CGRP was washed out after 5 min of exposure. Note the appearance of internalized CGRP receptors as early as 10 min after treatment onset and large endosome-like vesicles being present at 60 min after CGRP stimulation (arrowheads). Membrane staining returned after 5 h and was back to control levels after 24 h (**a**). Time course of sustained, 100 nM CGRP-induced internalization of CGRP (8E11, green) together with the CGRP receptor (RAMP1, red) over 24 h (**b**). Few double positive, yellow vesicles emerged as early as 10 min after treatment onset (arrowheads), increased in number at 60 min (arrowheads) and in size and labeling intensity at 5 and 24 h (**b**). Scale bars = 12 μm

In contrast to the persistent perinuclear staining in response to continuous exposure to CGRP, brief exposure to CGRP resulted in transient formation of AA58-, RAMP1-, and CLR-FAP-positive, endosome-like vesicles followed by reappearance of the staining at the plasma membrane (Fig. 6a). Transient, 5 min exposure to 100 nM CGRP followed by wash-out with fresh media resulted in initial formation of AA58-, RAMP1-, and CLR-FAP-positive endosome-like vesicles as early as 10 min after wash-out with a peak at 60 min (Fig. 6a). Reappearance of CGRP receptor immunoreactivity at the plasma membrane was observed as early as 2–3 h with parallel disappearance of endosome-like vesicular staining from the cytoplasm (not shown). At 5 h after the initial ligand pulse, immunostaining was mainly localized to the plasma membrane and was maintained there for at least up to 24 h, the last time point assessed (Fig. 6a). Cells remained healthy in this context as suggested by the healthy morphological appearance of the nuclei as assessed by Hoechst staining.

### Internalization of CGRP

After demonstrating that CLR and RAMP1 localize together during CGRP receptor internalization in the CHO cell model, we investigated whether CGRP also internalized and co-localized with the CGRP receptor (Fig. 6b). Using staining with the anti-CGRP monoclonal antibody 8E11 to detect CGRP, and RAMP1 staining to detect CGRP receptors (AA58 could not be used, since it is a protein from the same species as 8E11), we found that CGRP internalized and trafficked in a similar fashion as the CGRP receptor. CGRP receptor-bound CGRP at the plasma membrane could not be visualized by 8E11 at the 0 min time point (Fig. 6b). However, at 10 min after continuous CGRP exposure, 8E11 positive, endocytic vesicle-like structures appeared in the cytoplasm, many of which were also positive for RAMP1 (Fig. 6b). The co-localization became stronger at 60 min and was most pronounced in dense, perinuclear, presumably lysosomal structures at 5 h and 24 h, the last time point investigated (Fig. 6b).

### Inhibition of CGRP receptor internalization by monoclonal antagonist antibodies

**Fig. 7:**
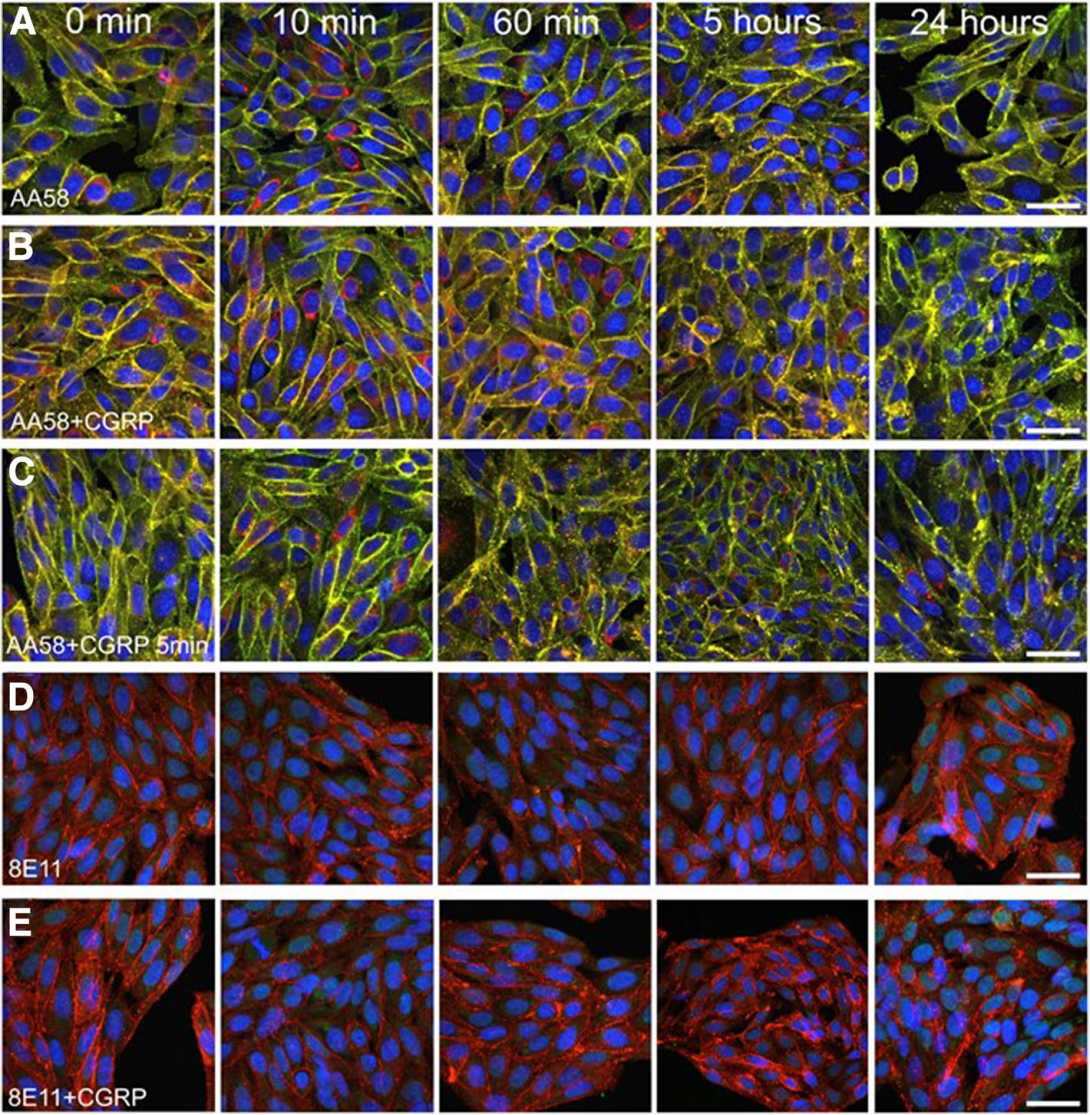
Inhibition of CGRP receptor internalization by monoclonal antagonist antibodies. Time course of triple positive (yellow) CGRP receptor (AA58 = green, RAMP1 = red, CLR-FAP = magenta) trafficking over 24 h (**a**-**c**): Treatment with 479 nM AA58 alone (**a**), in the presence of sustained exposure to 100 nM CGRP (**b**) and after 5 min exposure to CGRP followed by wash-out of both CGRP and unbound AA58 (**c**). Time course of CGRP receptor trafficking over 24 h visualized by RAMP1 (red, **d**, **e**): Treatment with 479 nM 8E11 alone (**d**) and with 8E11 in the presence of sustained 100 nM CGRP exposure (**e**). 8E11 (green) could not be visualized by secondary detection indicating that it had bound to CGRP and was removed during the fixation process (**d**, **e**). Scale bars = 12 μm

Both anti-CGRP receptor antagonist antibody AA58 or anti-CGRP antagonist antibody 8E11 did not have an effect in the absence of CGRP (Fig. 7a and d) but prevented CGRP-induced CGRP receptor internalization (Fig. 7b, c, and e). In the absence of CGRP, treatment of CHO cells with AA58 (70 μg/mL; 479 nM corresponding to >IC_90_ in the FACS experiment) did not induce internalization of CGRP receptors and AA58 (detected by secondary antibody), RAMP1, and CLR-FAP immunostaining remained co-localized at the plasma membrane at 0 min, 10 min, 60 min, 5 h and 24 h (Fig. 7a). Following co-treatment of CHO cells with AA58 (479 nM) and CGRP (100 nM), CGRP receptor immunostaining remained largely localized to the plasma membrane for up to 24 h, the last time point observed (Fig. 7b). The same effect was observed, when using the transient CGRP exposure paradigm for 5 min followed by a wash that would also remove unbound AA58 (Fig. 7c). A low level of endosome-like vesicle formation that stained triple positive for AA58, RAMP1 and CLR-FAP could be observed in the cytoplasm at all time points and appeared increased at the later time points (60 min to 24 h, Fig. 7b and c), most likely due to a residual effect of CGRP at the concentrations used. Similar to the lack of internalization observed with AA58 in the absence of CGRP, the anti-CGRP antibody 8E11 on its own did not cause CGRP receptor internalization as visualized by RAMP1 staining (Fig. 7d). Co-application of 8E11 (70 μg/ml; 479 nM, corresponding to the >IC_90_ in the FACS experiment) and 100 nM CGRP largely prevented CGRP-induced CGRP receptor internalization for up to 24 h (Fig. 7e). As with the CGRP receptor antibody, despite preservation of membrane labeling by RAMP1 with 8E11 treatment, a slight increase in endosome-like vesicle formation was also observed from 60 min to 24 h (Fig. 7e).

## Discussion

In the current study, we developed a CHO cell model and demonstrated that neither anti-CGRP (8E11) nor anti-CGRP receptor antibodies (AA58) have an effect in the absence of CGRP on cAMP accumulation or CGRP receptor internalization up to 24 h. Cell health as monitored by the integrity of nuclear staining, was not affected by these treatments during the 24 h of observation. The lack of an effect of the monoclonal antibodies in the absence of CGRP is consistent with data from experiments with the small molecule CGRP receptor antagonist telcagepant (MK-0974) in human arteries showing no effect of telcagepant under baseline conditions [25]. It is generally believed that antagonists do not promote G-protein coupled receptor (GPCR) internalization. However, paradoxical internalization by antagonists has been reported for some GPCRs, such as the serotonin 5-HT2A [26], neuropeptide Y Y1 [27], vasopressin V2 [28], angiotensin AT1 [29], and bradykinin B2 receptors [30]. Paradoxical internalization by antagonists had not been reported for CGRP receptors in line with our findings with antagonist antibodies. In humans, erenumab has demonstrated non-linear pharmacokinetics due to receptor mediated clearance at lower doses up to 70 mg [31]. This receptor mediated clearance is most likely due to the internalization of the antibody with the receptor (antibody-receptor complex) through its natural membrane trafficking cycle. This “internalization half-life” is roughly estimated based on pharmacokinetic parameters as 1–2 days [32]. However, it was not measured in the current study model, but was used as a baseline.

We also demonstrate how monoclonal antagonist antibodies targeting either CGRP or the CGRP receptor interfere with CGRP receptor signaling. The CHO cells stably expressing CLR-FAP tagged receptors that were used in our study behaved similar to cell lines reported in the literature in response to CGRP (reviewed in [33]). Application of CGRP resulted in a dose-dependent increase in cAMP production with an average EC_50_ of 8.4 pM. At nanomolar concentrations (average EC_50_ of 7.9 nM) CGRP caused CGRP receptor internalization in our cell line as shown by FACS. This shift in potency is likely not only assay-dependent, but also caused by temporal and functional differences in cAMP signaling compared to internalization pathways (for recent reviews, see [34–36]). The activation of different signaling pathways by different concentrations and temporal activation profiles of CGRP could be relevant for migraine pathophysiology [15]. In HEK293 cells, CGRP receptor internalization induced by CGRP (100 nM, the same concentration used for internalization in our study) has been shown to activate sustained endosomal signaling [15]. In our study, we saw that within 60 min of CGRP application, most internalized CGRP receptors localize to endosomes positive for EEA1. Yarwood et al. suggested that only the endosomal, not the plasma membrane signaling by CGRP mediates pain transmission: Blockade of endosomal CGRP-bound CGRP receptor signaling by the peptide antagonist CGRP_8–37_ conjugated to cholesterol for endosome-specific targeting resulted in inhibition of nociceptive responses to formalin injection in mice [15]. It could be hypothesized that higher levels of CGRP may contribute to migraine pain via CGRP receptor internalization and endosomal signaling. Future studies would be required to assess the relationship between endogenous concentrations of CGRP to CGRP receptor-mediated endosomal signaling and pain transmission during migraine attacks.

Using confocal microscopy we corroborated previous findings [37] that transient, in our experiments 5 min, exposure to CGRP at levels expected to activate most receptors (100 nM, EC_90_) results in recycling of internalized CGRP receptors to the cell surface within 24 h, whereas continuous exposure to the same amount of CGRP results in degradation of CGRP receptors indicated by the appearance of lysosomal marker LAMP2 and CGRP receptor double positive vesicles at 60 min with no recycling visible up to 24 h. Moreover, we confirmed that CGRP internalizes together with the CGRP receptor in our cell model [14, 38, 39].

In the presence of CGRP, anti-CGRP and anti-CGRP receptor antibodies blocked cAMP signaling at nanomolar concentrations with average IC_50_ values of approximately 1.7 nM and 0.9 nM, respectively. CGRP-induced receptor internalization is prevented by either of the antibodies at ten-fold higher concentrations with average IC_50_ values around 29 nM for anti-CGRP and 9 nM for anti-CGRP receptor antibodies. Whether the higher concentrations needed to block internalization (and subsequent endosomal signaling) as compared to cAMP accumulation has clinical meanings is yet to be seen. The potency difference between blocking the cAMP signaling and CGRP-induced receptor internalization may be due to the overexpression system in the current study wherein the rate-limiting machinery of internalization is out-numbered by the overwhelming amount of the receptors that are super reactive to the agonist. However, it is unknown whether the potency based on receptor internalization is more relevant to the clinical efficacy in a therapeutic setting. Both, anti-CGRP and anti-CGRP receptor antibodies blocked cAMP accumulation and CGRP receptor internalization in a comparable manner in our cell model. This cell model helped us to both measure and visualize the pharmacological effects of our tool antibodies but is not sufficient to make predictions for efficacy of one type of antibody over the other in patients. So far, all therapeutic antibodies targeting the CGRP pathway have shown efficacy in clinical trials [4, 5]. Our data from the cell model indicates that either mechanism, inactivating CGRP by anti-CGRP antibodies or blocking its access to the CGRP receptor by anti-CGRP receptor antibodies, interrupts CGRP-induced signaling via cAMP accumulation and inhibits CGRP receptor internalization. The different antibodies may have different effects in vivo that were not assessed in this study, given that CGRP can bind to other receptors (which would be affected by anti-ligand not anti-receptor antibodies). Future research directed towards investigating the different CGRP signaling pathways in trigeminal neurons and their target tissues may lead to further understanding of CGRP’s role in migraine pathophysiology [40, 41].

## Conclusions

In this study, we used tool monoclonal antagonist antibodies and a new cellular model to study CGRP receptor function and internalization by flow cytometry and confocal microscopy. The results demonstrate that functional anti-CGRP and anti-CGRP receptor antibodies do not impact CGRP receptor mediated cAMP accumulation and internalization unless CGRP is present. These data reinforce our understanding how monoclonal functional antagonist antibodies interfere with CGRP signaling.

## Abbreviations

CCLR: Calcitonin-receptor-like receptor
CGRP: Calcitonin gene-related peptide
CHO: Chinese hamster ovary
FAP: Fluorogen-activated protein
FBS: Fetal bovine serum
RAMP1: Receptor activity-modifying protein 1
